# An Upper Bound for Accuracy of Prediction Using GBLUP

**DOI:** 10.1371/journal.pone.0161054

**Published:** 2016-08-16

**Authors:** Emre Karaman, Hao Cheng, Mehmet Z. Firat, Dorian J. Garrick, Rohan L. Fernando

**Affiliations:** 1 Department of Animal Science, Faculty of Agriculture, Akdeniz University, 07059 Antalya, Turkey; 2 Department of Animal Science, Iowa State University, 50011 Ames, Iowa, United States of America; 3 Department of Statistics, Iowa State University, 50011 Ames, Iowa, United States of America; 4 Institute of Veterinary, Animal and Biomedical Science, Massey University, Palmerston North, New Zealand; Loyola University Chicago, UNITED STATES

## Abstract

This study aims at characterizing the asymptotic behavior of genomic prediction *R*^2^ as the size of the reference population increases for common or rare QTL alleles through simulations. Haplotypes derived from whole-genome sequence of 85 Caucasian individuals from the 1,000 Genomes Project were used to simulate random mating in a population of 10,000 individuals for at least 100 generations to create the LD structure in humans for a large number of individuals. To reduce computational demands, only SNPs within a 0.1M region of each of the first 5 chromosomes were used in simulations, and therefore, the total genome length simulated was 0.5M. When the genome length is 30M, to get the same genomic prediction *R*^2^ as with a 0.5M genome would require a reference population 60 fold larger. Three scenarios were considered varying in minor allele frequency distributions of markers and QTL, for *h*^2^ = 0.8 resembling height in humans. Total number of markers was 4,200 and QTL were 70 for each scenario. In this study, we considered the prediction accuracy in terms of an estimability problem, and thereby provided an upper bound for reliability of prediction, and thus, for prediction *R*^2^. Genomic prediction methods GBLUP, BayesB and BayesC were compared. Our results imply that for human height variable selection methods BayesB and BayesC applied to a 30M genome have no advantage over GBLUP when the size of reference population was small (<6,000 individuals), but are superior as more individuals are included in the reference population. All methods become asymptotically equivalent in terms of prediction *R*^2^, which approaches genomic heritability when the size of the reference population reaches 480,000 individuals.

## Introduction

The availability of single nucleotide polymorphism (SNP) marker chips for many species has given rise to the era of genomic prediction (GP). As the name suggests, GP refers to the use of genomic information to predict genetic merit and can be applied to animals [1–4], plants [5–7], or to predisposition to disease in personalized medicine [8]. GP utilizes the phenotypes and SNP genotypes of a group of individuals (hereafter, called the reference population-RP) to estimate marker effects, which are used to predict breeding values, or yet-to-be observed phenotypes of individuals with genotypes (hereafter called the validation population-VP) [1].

The accuracy of GP is influenced by many factors, such as the method used to estimate marker effects [9, 10], the heritability (*h*^2^) and genetic architecture of the trait [10, 11], and the size (*n*_*R*_) and structure of the RP [11–16]. Among those, the method, and the size and structure of the RP can be chosen or designed utilizing available knowledge about the heritability and genetic architecture of the trait.

One of the challenges of GP using high-density SNP genotypes is the estimation of SNP effects when the number of individuals comprising RP, *n*_*R*_, is much smaller than the number of SNPs *p*, *n*_*R*_ ≪ *p*. One approach to address this problem is Bayesian regression, which combines prior information on the vector of SNP effects, ***β***, with the observed data to estimate all *p* of the *β*_*j*_s [1]. There are several variations of the Bayesian regression approach, differing in the prior distribution for *β*_*j*_. A commonly used prior for *β*_*j*_ is a normal distribution with a common variance for all loci: $\beta_{j}∼N\left(0,\;\sigma_{\beta }^{2}\right).$ This is equivalent to ridge regression, and when the ratio $\sigma_{\beta }^{2}/\sigma_{e}^{2}$ is known, it can be shown that it becomes best linear unbiased prediction (BLUP) [17]. Due to the relationship of this Bayesian regression to ridge regression and BLUP, it is referred to as Bayesian ridge regression (BRR) or random regression BLUP (RR-BLUP). Genomic predictions obtained from RR-BLUP are identical to those obtained from an animal model (GBLUP), where the numerator relationship matrix is replaced by a genomic relationship matrix (**G**) computed from markers [9, 18–21].

The GBLUP method is popular in GP for three reasons: 1) Since for several decades selection decisions in livestock populations have been routinely made based on BLUP for the animal model [22], GBLUP can easily be used with current computer programs without much effort, 2) for BLUP, theory is available to compute the variance of prediction errors, and 3) many important traits in animals, plants and humans are complex in nature, and are controlled by a large number of small effect genes distributed across the entire genome [23–25], resembling an infinitesimal model [1]. When this assumption does not hold, because a few genes have large effects, or because many genes have no effect, mixture models such as BayesB [1] or BayesC [26, 27] can be used, where the prior for *β*_*j*_ has a point mass at zero with probability *π*, or has a *t* or normal distribution with probability (1 − *π*) for BayesB and BayesC, respectively.

The squared correlation between the genetic value (*u*) and its predicted value ($\hat{u}$) is called the reliability of prediction, and is a measure of prediction accuracy. Goddard [16] and Daetwyler et al. [28] have developed approximations for prediction accuracy utilizing the effective population size (*N*_*e*_), *n*_*R*_, *h*^2^, and the effective number of chromosomal segments segregating in the population (*M*_*e*_). Both approximations were developed assuming complete linkage disequilibrium (LD) between marker-QTL pairs. Goddard et al. [29] extended his earlier approach to address the problem of incomplete LD between markers and QTL. Following that extension, reliability of GP can be approximated with *q*^2^[*n*_*R*_ *h*^2^/(*n*_*R*_ *h*^2^ + *M*_*e*_/*q*^2^)], where *q*^2^ is the proportion of genetic variance explained by markers. In contrast to simulations, in real applications *u* of an individual is not observed, and therefore, reliability of prediction cannot be directly computed. Thus, the squared correlation between phenotype (*y*) and $\hat{u}$ (hereafter, called the *R*^2^) is often used as the measure of prediction accuracy. The approximation for reliability in [29] can be modified (See Appendix 1 in S1 Text) to get an approximation for *R*^2^ as:

$$\begin{array}{c} R^{2}\approx h_{M}^{2}\left[n_{R}h_{M}^{2}/\left(n_{R}h_{M}^{2}+M_{e}\right)\right], \end{array}$$ (1)

where $h_{\text{M}}^{2}$ is the genomic heritability, the proportion of variance explained by markers [30].

In pedigree-based prediction, heritability is a major determinant of *R*^2^. Using both real data and simulations based on real genotypes, de los Campos et al. [30] investigated the relationship between $h_{\text{M}}^{2}$ and *R*^2^ from GBLUP for complex traits in humans. They examined different scenarios varying in the distribution of minor allele frequencies (MAFs) of markers and QTL. When both RP and VP included only unrelated individuals, *R*^2^ and $h_{\text{M}}^{2}$ were 0.071 and 0.737 when markers and QTL had similar MAF distributions, and 0.049 and 0.573 when MAF for QTL were low relative to those for markers. It was concluded that $h_{\text{M}}^{2}$ is not a good indicator of *R*^2^ when the individuals being predicted are not related to the RP. Instead, the authors proposed [1 − (1 − *b*)^2^]*h*^2^ as an upper bound for *R*^2^, where *b* is the average regression coefficient of the marker derived relationships on QTL derived relationships. Based on the average value of *b* estimated for candidates unrelated to the reference population, they concluded that the asymptotic upper bound on the *R*^2^ is about 20% of *h*^2^ for unrelated individuals. This conflicts with approximation Eq (1) where it can be seen that the asymptotic value for *R*^2^ is predicted to be $h_{\text{M}}^{2}$.

Taking *N*_*e*_ = 10,000 [31], the average chromosome length (*L*) as 1.57 Morgans, and the number of chromosomes (*k*) as 23, to represent humans, $M_{e}=\frac{2N_{e}Lk}{log(N_{e}L)}\approx 7\times 10^{4}$ [16], and then from Eq (1) the expected *R*^2^ is 0.037 for $h_{M}^{2}=0.737$ and 0.022 for $h_{M}^{2}=0.573$ when *n*_*R*_ = 5,300 as in [30]. These are close to the *R*^2^ values in [30] for unrelated individuals. In order for *R*^2^ to reach about 90% of $h_{\text{M}}^{2}$, approximation Eq (1) suggests that a RP of over half a million individuals is needed. Thus, computer simulation will be used to examine whether the upper bound in [30] holds or if *R*^2^ reaches $h_{\text{M}}^{2}$ when *n*_*R*_ increases as implied by Eq (1). Further, the suggestion in [30] that variable selection methods may have higher accuracy of prediction than GBLUP for complex traits in humans is examined.

There is growing interest on the optimum structure of the RP [13, 32], accounting for its impact on the relationship between VP and RP individuals [9, 12, 13, 15, 33, 34]. On the other hand, the definition of relatedness in most studies is based on concepts associated with pedigrees, which depend on how deep the pedigree is traced and which measure of relationship is used (e.g. average squared relationship, mean relationship). However, the **G** matrix better reflects genetic similarities between individuals than the numerator relationship matrix computed from pedigree. When the main interest is to rank individuals in the VP according to their predicted genetic values, $\hat{u}$, the use of pairwise relationships between VP and RP can be misleading. In this article, we approach GP as an estimability problem and accordingly provide an upper bound for reliability of prediction, and therefore an upper bound for prediction *R*^2^.

## Materials and Methods

### Data Sets and Simulation of Genomes

The central objective of this study was to examine the asymptotic behavior of *R*^2^, which requires a large RP. To simulate genotypes that resembles the LD structure in humans for a large number of individuals, haplotypes of 85 Caucasian individuals from the 1,000 Genome Project [35] (ftp://ftp.1000genomes.ebi.ac.uk/vol1/ftp/release/20110521/) were used to generate a population of size 10,000. This was accomplished by randomly sampling 20,000 gametes from phased paternal and maternal sequence accounting for crossing over and mutation. Following this, 10,000 offspring were sampled from random mating of 10,000 parents for 111 non-overlapping generations. Generations 101 and 111 were used to form RP and VP, respectively. A mutation rate of 1 × 10^−8^ was used. Mutations switched the original allele state from 0 to 1, or vice versa. Simulation of the genomes were performed using XSim software, which uses an algorithm that tracks only the positions of crossing over and mutation as well as the origin of each chromosomal segment throughout the generations, and finally, drops sequence variants from the founders to the individuals in the last generation [36].

To reduce computational demands, only SNPs with known positions within a 0.1M region of each of the first 5 chromosomes were used. Markers with MAF <0.005 were discarded resulting in a data set including 36,242 SNPs. To make our data set comparable with [30], we scaled down these loci to 4,200 to be used as markers and an additional 70 to represent QTL. Hence, the density of markers (84/cM) and ratio of the number of QTL (*n*_*QTL*_) to markers (1/6) are similar to those in [30] that was based on 300,000 markers and 5,000 QTL. Three different scenarios (SHL-SRR) were created varying in the MAF distributions: (SHL) markers and QTL were selected for high and low MAFs, respectively; (SHR) markers were selected for high MAF, whereas QTL were selected at random; and (SRR) markers and QTL were selected at random. Hence, among the 36,242 SNPs, 4,200 were taken to be markers, and 70 as QTL among those with high, random or low MAF as appropriate. Scenario SHL corresponds to Low-MAF, whereas SHR corresponds to RAND in [30]. The MAF distributions for the three scenarios are summarized in Table 1. Different MAF scenarios allowed us to examine the impact of the structure of SNP chips on accuracy.

**Table 1:** Percentage of SNPs in a particular range of MAF.

Scenario	MAF	Type	<3%	3%-5%	5%-10%	10%-15%	>15%
SHL	High	Marker	0.061	0.046	0.117	0.114	0.663
	Low	QTL	0.314	0.243	0.229	0.200	0.014
SHR	High	Marker	0.065	0.049	0.119	0.115	0.652
	Random	QTL	0.029	0.057	0.100	0.100	0.714
SRR	Random	Marker	0.082	0.050	0.096	0.104	0.668
	Random	QTL	0.100	0.029	0.114	0.114	0.643

MAF: minor allele frequency; SHL: markers and QTL were selected for high and low MAFs, respectively; SHR: markers were selected for high MAF, whereas QTL were selected at random; SRR: markers and QTL were selected at random

### Reference and Validation Populations

In this study, RP and VP were separated for 10 generations to ensure that close relatives of the VP individuals do not exist in RP. Both generations consisted of 10,000 individuals. Among the individuals in generation 101, *n*_*R*_ individuals were randomly selected to form a RP, for a range of *n*_*R*_ (75, 150, 500, 1,000, 2,000, 4,000 and 8,000), while *n*_*V*_ = 2,000 individuals were selected among the individuals in generation 111 to form a VP.

### Simulation of Phenotypes

Similar to de los Campos et al. [30], a quantitative trait corresponding to human height with heritability 0.8 was simulated. The effects of QTL, *α*_*j*_s, were sampled from a normal distribution, *α*_*j*_ ∼ *N*(0, 1). Since the QTL effect sizes vary by replicate, the genetic variance can vary by replicate. To keep the heritability constant across replicates, therefore, QTL effects were scaled at each replicate. The product of the scaled QTL effects and the QTL genotypes was used to obtain the genetic value of individual *i* as follows:

$$\begin{array}{c} u_{i}=\sum_{j=1}^{n_{QTL}}\alpha_{j}\times Q_{ij}, \end{array}$$

where *n*_*QTL*_ is the number of QTL, *α*_*j*_ is the additive effect of *j*’th QTL, and *Q*_*ij*_ is the genotype of individual *i* at the *j*’th QTL. In each replicate of each scenario, the same SNPs were designated as markers or QTL, however, QTL effects were separately randomly simulated in each replicate. A standard normal deviate (*e*_*i*_) was added to each individual’s *u*_*i*_ to form its phenotypic value (*y*_*i*_) with the desired heritability.

### Estimation of Marker Effects

The statistical model fitted to the data is:

$$\begin{array}{c} y_{i}=\mu +\sum_{j=1}^{p}x_{ij}\beta_{j}+e_{i}, \end{array}$$

where *y*_*i*_ is the phenotypic value of individual *i* in the RP, *μ* is the overall mean, *p* is the number of marker loci, *x*_*ij*_ is the marker genotype of individual *i* at locus *j*, *β*_*j*_ is the allele substitution effect of marker *j*, and *e*_*i*_ is the random environmental effect assumed to be normally distributed, $e_{i}∼N\left(0,\;\sigma_{e}^{2}\right)$.

To predict the genetic value of individuals, marker effects were first estimated from RP data using BayesB and BayesC methods, which differ in the prior assumptions for marker effects as described previously. BayesC with *π* = 0 is identical to GBLUP when $\sigma_{\beta }^{2}$ is treated as unknown with a scaled inverse chi-square prior. In the BayesB and BayesC analyses, *π* was 0.98, whereas GBLUP results were obtained using BayesC with *π* = 0. A total of 11,000 Markov chain Monte Carlo iterations were used, with the first 1,000 excluded as the burn-in. Marker effects were estimated from separate analyses with inclusion or exclusion of QTL from the marker panel. Analyses were performed using GenSel software [37, 38]. In order to evaluate how frequently a marker was included in the model in a BayesB or BayesC run, the model frequency (MF) in GenSel output can be used which is defined as the proportion of iterations or models that included that marker.

### Prediction of Genetic Values, and Prediction Accuracy

Given the estimates of the marker effects, the *u* of the VP individuals was predicted as:

$$\begin{array}{c} {\hat{u}}_{i}=\sum_{j=1}^{p}x_{ij}\hat{\beta_{j}} \end{array}$$

where ${\hat{\beta }}_{j}$ is the estimated effect of locus *j*, and *x*_*ij*_ is the marker genotype of *i*’th individual at locus *j*. The prediction *R*^2^ was calculated as the squared correlation between the phenotypes, *y*_*i*_, and ${\hat{u}}_{i}$ of VP individuals.

Using the regression model:

$$\begin{array}{c} {\text{g}}_{M,i}=b_{i}\times {\text{g}}_{Q,i}+\epsilon_{i} \end{array}$$ (2)

where **g**_*M*,*i*_ is the vector of marker derived relationships of *i*’th individual in VP to all the individuals in RP, and **g**_*Q*,*i*_ is the vector of QTL derived relationships, and *ϵ*_*i*_ is a vector of residuals, the regression coefficient, *b*_*i*_, was estimated [30] and averaged across all individuals in VP for each replicate. Both forms of relationships were obtained from the realized relationship matrix, **G**:

$$\begin{array}{c} \text{G}=\frac{\text{Z}{\text{Z}}^{\prime }}{p} \end{array}$$

where **Z** is the matrix of genotypes constructed by the standardized vector of (marker or QTL) genotypes including RP and VP, and *p* is the number of loci (marker or QTL) [25]. Standardization of genotypes was done as follows, where **x**_*j*_ is a vector of genotypes of individuals at *j*’th loci, and *q*_*j*_ is the allele frequency:

$$\begin{array}{c} {\text{z}}_{j}=\frac{{\text{x}}_{j}-2q_{j}}{\sqrt{2q_{j}\left(1-q_{j}\right)}}. \end{array}$$

Scenarios SHL, SHR, and SRR, were replicated 10 times, and results were averaged across replicates.

### A Connection between Prediction Accuracy and Estimability in a Fixed Linear Model

Consider the linear model:

$$\begin{array}{c} \text{y}=\text{X}\beta +\text{e}, \end{array}$$

where ***β*** is assumed fixed. In this setting, a linear function, **k**′ ***β*** is said to estimable only if the estimator ${\text{k}}^{\prime }\hat{\beta }$ has expected value **k**′ ***β***. When $\hat{\beta }$ is the least-squares estimator, it is known that **k**′ ***β*** is estimable only when **k**′ is a linear function of the rows of **X** [39].

In RR-BLUP, ***β*** is considered random with null mean, and the BLUP of ${\text{x}}_{V}^{\prime }\beta$ representing an individual in VP with genotypes **x**_*V*_ is unbiased in the sense that

$$\begin{array}{c} E\left({\text{x}}_{V}^{\prime }\tilde{\beta }\right)=E\left({\text{x}}_{V}^{\prime }\beta \right)=0, \end{array}$$

where $\tilde{\beta }$ is the BLUP of ***β***. To see the connection between prediction accuracy and estimability, let **X**_*R*_ be the genotype matrix of RP and let ℜ(**X**_*R*_) denote the row space of **X**_*R*_. Then, any vector **x**_*V*_ can be written as the sum of two vectors: ${\text{x}}_{V_{1}}={\text{Q}}_{X_{R}^{\prime }}{\text{x}}_{V}$, which is in ℜ(**X**_*R*_), and ${\text{x}}_{V_{2}}=\left(\text{I}-{\text{Q}}_{X_{R}^{\prime }}\right){\text{x}}_{V}$, which is orthogonal to ℜ(**X**_*R*_), i.e., the vector of validation genotypes can be written as

$$\begin{array}{c} {\text{x}}_{V}={\text{x}}_{V_{1}}+{\text{x}}_{V_{2}}, \end{array}$$ (3)

where ${\text{Q}}_{X_{R}^{\prime }}={\text{X}}_{R}^{\prime }{\left({\text{X}}_{R}{\text{X}}_{R}^{\prime }\right)}^{-}{\text{X}}_{R}$ [39]. From Eq (3), ${\text{x}}_{V}^{\prime }\tilde{\beta }$ of an individual in VP can be partitioned as:

$$\begin{array}{c} {\text{x}}_{V}^{\prime }\tilde{\beta }={\text{x}}_{V_{1}}^{\prime }\tilde{\beta }+{\text{x}}_{V_{2}}^{\prime }\tilde{\beta }. \end{array}$$ (4)

It is shown in Appendix 2 of S1 Text that $\tilde{\beta }$ is in ℜ(**X**_*R*_), and therefore, the BLUP of $\text{x}{}_{V}^{\prime }\beta$ is

$$\begin{array}{ccc} {\text{x}}_{V}^{\prime }\tilde{\beta } & = & \text{x}{}_{V_{1}}^{\prime }\tilde{\beta }. \end{array}$$ (5)

Accordingly, ${\text{x}}_{V_{2}}^{\prime }\tilde{\beta }=0,$ which is the mean of its prior and does not depend on the data. This can be seen more clearly by writing the correlation between $u_{i}={\text{x}}_{V}^{\prime }\beta$ and ${\hat{u}}_{i}=\text{x}{}_{V}^{\prime }\tilde{\beta }$ in terms of Eq (5) as shown below. Under BLUP assumptions [22],

$$\begin{array}{ccc} Cor(u_{i},{\hat{u}}_{i}) & = & \sqrt{\frac{Var({\hat{u}}_{i})}{Var(u_{i})},} \end{array}$$ (6)

and from Eq (5), the numerator of Eq (6) becomes $Var({\text{x}}_{V_{1}}^{\prime }\tilde{\beta }).$ So, Eq (6) can be written as

$$\begin{array}{c} Cor\left(u_{i},{\hat{u}}_{i}\right)=\sqrt{\frac{Var({\text{x}}_{V_{1}}^{\prime }\tilde{\beta })}{Var({\text{x}}_{V}^{\prime }\beta )}.} \end{array}$$

Clearly, $Var({\text{x}}_{V_{2}}^{\prime }\tilde{\beta })=0$ and does not contribute to $Cor(u_{i},{\hat{u}}_{i})$. An individual for whom the genotype vector is orthogonal to all genotypes in RP (**x**_*V*_ = **x**_*V*_2__) can be thought of as being genomically unrelated to the RP. For such an individual, $Cor(u_{i},{\hat{u}}_{i})$ would be zero. On the other hand, an individual for whom the genotype vector is in the row space of genotypes in RP (**x**_*V*_ = **x**_*V*_1__) can be thought of as having a perfect genomic relationship to the RP. This is not a genomic relationship between two individuals, and it does not require a perfect or even high relationship with any individual in RP. For such an individual, $Cor(u_{i},{\hat{u}}_{i})$ will approach 1 as $Var({\text{x}}_{V_{1}}^{\prime }\tilde{\beta })$ approaches $Var({\text{x}}_{V}^{\prime }\beta ).$ Generally, the maximum value of $Cor(u_{i},{\hat{u}}_{i})$ is $\sqrt{\frac{Var({\text{x}}_{V_{1}}^{\prime }\beta )}{Var({\text{x}}_{V}^{\prime }\beta )}},$ which is the square root of reliability defined as $Cor^{2}\left(u_{i},{\hat{u}}_{i}\right)$. Thus

$$\begin{array}{c} UP_{i}=\frac{{\text{x}}_{V_{1}}^{\prime }{\text{x}}_{V_{1}}}{{\text{x}}_{V}^{\prime }{\text{x}}_{V}} \end{array}$$

is a measure of the upper bound for reliability (See Appendix 3 in S1 Text). When *UP*_*i*_ = 0, the reliability of prediction will be zero regardless of the size of RP, and when *UP*_*i*_ = 1, the reliability will approach 1 as the size of RP increases. On the other hand when *UP*_*i*_ < *a*, reliability will be less than *a* regardless of the size of the RP. In addition, *h*^2^ *UP*_*i*_ is the upper bound of the *R*^2^ for individual *i*.

In order to examine the utility of this upper bound, we carried out a simulation study randomly selecting 5,000 of the 36,242 available SNPs. All of the selected SNPs were used as QTL, but two quantitative traits were simulated representing *h*^2^ = 0.8 or 0.999. Among the 10,000 individuals in generation 101, *n*_*R*_ individuals were randomly selected to form a RP, where *n*_*R*_ was varied (500, 1,000, 2,000 and 5,000), while *n*_*V*_ = 2,000 individuals were selected among the individuals of generation 111 to form a VP. The GBLUP method was used to predict genetic values, again through BayesC with *π* = 0. Other steps of the analysis were the same as given before. Setting *h*^2^ = 0.999, allowed us to minimize the estimation error of marker effects, so that prediction accuracy was determined almost entirely by estimability of the genotypes of VP. Effect of relationship to the RP for VP individuals was investigated utilizing the maximum relationships only at QTL level, *max*(**g**_*V*_*i*__), of individual *i* in VP to the individuals in RP. Individuals in VP were classified into a low (L) and high (H) relationship group: when an individual’s maximum relationship was lower than 0.15, it was assigned to L, while an individual with maximum relationship equal or greater than 0.25 was assigned to H.

## Results and Discussion

### *R*^2^ for GBLUP

**Fig 1:**
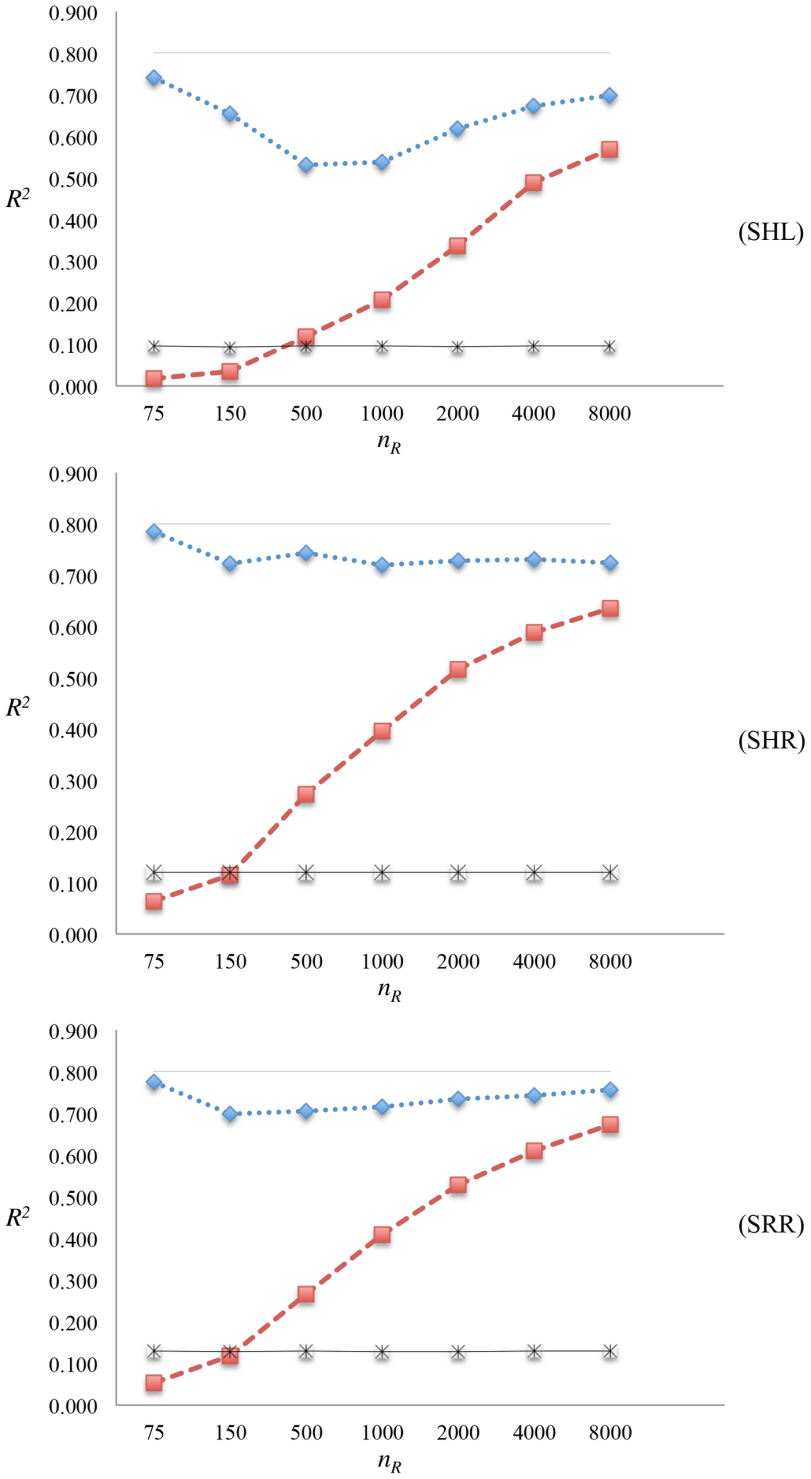
Summary of GBLUP results. SHL: markers and QTL were selected for high and low MAFs, respectively; SHR: markers were selected for high MAF, whereas QTL were selected at random; SRR: markers and QTL were selected at random; *n*_*R*_: number of individuals in RP; *R*^2^: squared correlation between *y* and $\hat{u}$; prediction *R*^2^ of GBLUP (red line); genomic heritability, $h_{\text{M}}^{2}$ (blue line); simulated heritability, $h_{sim}^{2}$ (grey line); the upper bound suggested in [30], [1 − (1 − *b*)^2^]*h*^2^ (black line).

Prediction *R*^2^s obtained using the GBLUP method with markers only are summarized in Fig 1 for varying RP sizes and all the scenarios along with the estimated heritabilities and the upper bound suggested by [30]. It is clear from the figure that an increase in the number of individuals in RP results in an increase in prediction *R*^2^ in all the scenarios. This is true even when the markers and QTL have an opposite MAF distribution (SHL), which may be the case in real data studies. In SHL, when RP involves only 75 individuals *R*^2^ was 0.017, while a maximum *R*^2^ of 0.569 was obtained for the largest RP size of 8,000 (Table 2). In SHR, where the markers had high MAF distribution, and QTL were selected completely at random, a RP size of 75 resulted an *R*^2^ of 0.064, while a RP of size 8,000 yielded the highest *R*^2^ of 0.635 (Table 3). When the markers and QTL were selected completely at random, *R*^2^s were higher than their counterparts in SHL (Table 4). The lowest and highest *R*^2^ values in SRR were 0.053 and 0.673, for a RP size of 75 and 8,000, respectively.

**Table 2:** Results of SHL.

Method	*n*_*R*_	*n*_*V*_	*R*^2^	$h_{\text{M}}^{2}$	$R_{M+QTL}^{2}$	$h_{M+QTL}^{2}$
GBLUP	75	2,000	0.017(0.004)	0.741(0.021)	0.023(0.005)	0.686(0.027)
BayesB	75	2,000	0.032(0.007)	0.731(0.013)	0.069(0.016)	0.770(0.015)
BayesC	75	2,000	0.027(0.005)	0.708(0.027)	0.052(0.012)	0.722(0.030)
GBLUP	150	2,000	0.035(0.004)	0.654(0.030)	0.046(0.005)	0.661(0.029)
BayesB	150	2,000	0.082(0.015)	0.664(0.021)	0.174(0.022)	0.730(0.019)
BayesC	150	2,000	0.062(0.011)	0.650(0.032)	0.130(0.016)	0.721(0.027)
GBLUP	500	2,000	0.119(0.011)	0.531(0.037)	0.159(0.011)	0.618(0.035)
BayesB	500	2,000	0.371(0.021)	0.605(0.018)	0.660(0.009)	0.788(0.007)
BayesC	500	2,000	0.382(0.026)	0.635(0.019)	0.683(0.008)	0.801(0.007)
GBLUP	1,000	2,000	0.207(0.010)	0.539(0.021)	0.283(0.009)	0.640(0.017)
BayesB	1,000	2,000	0.486(0.018)	0.604(0.017)	0.749(0.004)	0.794(0.003)
BayesC	1,000	2,000	0.492(0.019)	0.610(0.015)	0.755(0.003)	0.795(0.003)
GBLUP	2,000	2,000	0.336(0.012)	0.617(0.010)	0.465(0.008)	0.734(0.008)
BayesB	2,000	2,000	0.571(0.011)	0.628(0.008)	0.784(0.002)	0.801(0.002)
BayesC	2,000	2,000	0.573(0.011)	0.629(0.007)	0.786(0.002)	0.801(0.002)
GBLUP	4,000	2,000	0.489(0.008)	0.673(0.006)	0.619(0.002)	0.773(0.002)
BayesB	4,000	2,000	0.602(0.009)	0.652(0.007)	0.789(0.003)	0.802(0.001)
BayesC	4,000	2,000	0.599(0.009)	0.649(0.007)	0.789(0.003)	0.801(0.001)
GBLUP	8,000	2,000	0.569(0.008)	0.698(0.005)	0.702(0.002)	0.792(0.001)
BayesB	8,000	2,000	0.624(0.008)	0.659(0.006)	0.794(0.002)	0.801(0.001)
BayesC	8,000	2,000	0.622(0.008)	0.656(0.006)	0.794(0.002)	0.801(0.001)

SHL: markers and QTL were selected for high and low MAFs, respectively; *n*_*R*_: number of individuals in RP; *n*_*V*_: number of individuals in VP; *R*^2^ and $h_{\text{M}}^{2}$ are the predictive ability and genomic heritability when QTL are not in the panel; $R_{M+QTL}^{2}$ and $h_{M+QTL}^{2}$ are the predictive ability and genomic heritability when QTL are in the panel

**Table 3:** Results of SHR.

Method	*n*_*R*_	*n*_*V*_	*R*^2^	$h_{\text{M}}^{2}$	$R_{M+QTL}^{2}$	$h_{M+QTL}^{2}$
GBLUP	75	2,000	0.064(0.008)	0.784(0.011)	0.087(0.009)	0.757(0.013)
BayesB	75	2,000	0.051(0.005)	0.763(0.014)	0.092(0.014)	0.790(0.013)
BayesC	75	2,000	0.065(0.008)	0.754(0.015)	0.106(0.015)	0.775(0.014)
GBLUP	150	2,000	0.115(0.012)	0.722(0.028)	0.155(0.014)	0.750(0.023)
BayesB	150	2,000	0.207(0.020)	0.708(0.019)	0.298(0.021)	0.775(0.018)
BayesC	150	2,000	0.209(0.020)	0.729(0.027)	0.296(0.023)	0.793(0.022)
GBLUP	500	2,000	0.272(0.011)	0.743(0.013)	0.353(0.010)	0.819(0.009)
BayesB	500	2,000	0.473(0.014)	0.681(0.005)	0.682(0.009)	0.805(0.007)
BayesC	500	2,000	0.473(0.014)	0.692(0.005)	0.683(0.009)	0.808(0.007)
GBLUP	1,000	2,000	0.395(0.007)	0.719(0.012)	0.494(0.006)	0.803(0.008)
BayesB	1,000	2,000	0.569(0.009)	0.673(0.010)	0.754(0.003)	0.804(0.003)
BayesC	1,000	2,000	0.571(0.009)	0.675(0.010)	0.753(0.003)	0.803(0.003)
GBLUP	2,000	2,000	0.516(0.013)	0.728(0.008)	0.616(0.008)	0.802(0.004)
BayesB	2,000	2,000	0.629(0.011)	0.691(0.008)	0.780(0.003)	0.804(0.002)
BayesC	2,000	2,000	0.629(0.011)	0.691(0.008)	0.780(0.003)	0.803(0.002)
GBLUP	4,000	2,000	0.588(0.007)	0.730(0.005)	0.686(0.003)	0.800(0.002)
BayesB	4,000	2,000	0.655(0.009)	0.697(0.006)	0.789(0.002)	0.802(0.001)
BayesC	4,000	2,000	0.654(0.008)	0.696(0.006)	0.789(0.002)	0.802(0.001)
GBLUP	8,000	2,000	0.635(0.016)	0.724(0.011)	0.739(0.003)	0.800(0.001)
BayesB	8,000	2,000	0.665(0.018)	0.691(0.013)	0.796(0.002)	0.800(0.001)
BayesC	8,000	2,000	0.665(0.018)	0.690(0.013)	0.796(0.002)	0.800(0.001)

SHR: markers were selected for high MAF, whereas QTL were selected at random; *n*_*R*_: number of individuals in RP; *n*_*V*_: number of individuals in VP; *R*^2^ and $h_{\text{M}}^{2}$ are the predictive ability and genomic heritability when QTL are not in the panel; $R_{M+QTL}^{2}$ and $h_{M+QTL}^{2}$ are the predictive ability and genomic heritability when QTL are in the panel

**Table 4:** Results of SRR.

Method	*n*_*R*_	*n*_*V*_	*R*^2^	$h_{\text{M}}^{2}$	$R_{M+QTL}^{2}$	$h_{M+QTL}^{2}$
GBLUP	75	2,000	0.053(0.008)	0.775(0.009)	0.072(0.010)	0.748(0.009)
BayesB	75	2,000	0.073(0.016)	0.768(0.009)	0.124(0.021)	0.789(0.008)
BayesC	75	2,000	0.077(0.015)	0.753(0.010)	0.123(0.021)	0.774(0.009)
GBLUP	150	2,000	0.117(0.012)	0.698(0.041)	0.157(0.013)	0.724(0.037)
BayesB	150	2,000	0.187(0.027)	0.724(0.028)	0.304(0.024)	0.799(0.018)
BayesC	150	2,000	0.190(0.026)	0.731(0.044)	0.307(0.024)	0.814(0.023)
GBLUP	500	2,000	0.266(0.011)	0.705(0.014)	0.345(0.011)	0.790(0.009)
BayesB	500	2,000	0.501(0.017)	0.672(0.010)	0.691(0.006)	0.796(0.006)
BayesC	500	2,000	0.499(0.016)	0.682(0.010)	0.691(0.007)	0.800(0.006)
GBLUP	1,000	2,000	0.409(0.009)	0.716(0.017)	0.505(0.008)	0.801(0.011)
BayesB	1,000	2,000	0.588(0.008)	0.679(0.013)	0.760(0.004)	0.803(0.007)
BayesC	1,000	2,000	0.587(0.008)	0.682(0.013)	0.761(0.004)	0.803(0.007)
GBLUP	2,000	2,000	0.528(0.011)	0.735(0.009)	0.624(0.006)	0.803(0.004)
BayesB	2,000	2,000	0.647(0.013)	0.699(0.010)	0.784(0.002)	0.803(0.002)
BayesC	2,000	2,000	0.647(0.013)	0.698(0.010)	0.784(0.002)	0.802(0.002)
GBLUP	4,000	2,000	0.609(0.007)	0.743(0.005)	0.693(0.004)	0.800(0.001)
BayesB	4,000	2,000	0.678(0.007)	0.709(0.006)	0.792(0.002)	0.801(0.001)
BayesC	4,000	2,000	0.678(0.007)	0.708(0.005)	0.792(0.002)	0.801(0.001)
GBLUP	8,000	2,000	0.673(0.005)	0.757(0.003)	0.740(0.003)	0.801(0.001)
BayesB	8,000	2,000	0.706(0.006)	0.729(0.004)	0.795(0.002)	0.801(0.001)
BayesC	8,000	2,000	0.705(0.005)	0.728(0.004)	0.795(0.002)	0.801(0.001)

SRR: markers and QTL were selected at random; *n*_*R*_: number of individuals in RP; *n*_*V*_: number of individuals in VP; *R*^2^ and $h_{\text{M}}^{2}$ are the predictive ability and genomic heritability when QTL are not in the panel; $R_{M+QTL}^{2}$ and $h_{M+QTL}^{2}$ are the predictive ability and genomic heritability when QTL are in the panel

Heritability estimates were volatile in SHL, but almost flat in SHR and SRR. Moreover, the estimates of heritability were always greater for SHR and SRR than for SHL. In all scenarios, the *R*^2^ of GBLUP increased towards $h_{\text{M}}^{2}$. It is very likely that when sequence data are used, the fitted genotypes include the QTL. This motivated analyses with inclusion of the QTL genotypes in the marker panel, and even in that case, the predictive accuracy of GBLUP could not attain the estimated heritability for RP sizes considered here. However, the trend suggests that the predictive accuracy of GBLUP could achieve the heritability at a sufficiently large RP size. Regression coefficients for the marker derived relationships on the QTL derived relationships were obtained for every individual in VP, so that the upper bound for prediction *R*^2^, [1 − (1 − *b*)^2^]*h*^2^, suggested by [30] could be plotted for varying RP sizes as in Fig 1. The average of the regression coefficients (results not given), *b*, were almost invariant to RP size (varying only after 2nd digit), thereby yielding an upper bound that was invariant despite the predictive accuracy increasing with RP size. These results together demonstrate [1 − (1 − *b*)^2^]*h*^2^ is not an upper bound for *R*^2^ as claimed in [30]. When a sufficient number of individuals is in RP, predictive accuracy of GBLUP can reach $h_{\text{M}}^{2}$.

Using the Formula (1), the asymptotic value of *R*^2^ reaches the genomic heritability. In this simulation study, the predicted values of *R*^2^ for SHL-SRR from Eq (1) are 0.554, 0.579 and 0.611 with *n*_*R*_ = 8,000, $M_{e}=\frac{2N_{e}Lk}{log(N_{e}L)}=1,448$, for $h_{\text{M}}^{2}$ of 0.698, 0.724, 0.757, respectively. These predicted values are lower than the predictive accuracies using only markers (0.569, 0.635 and 0.673 for SHL-SRR, respectively), or the predictive accuracies when QTL were also in the panel (0.702, 0.739 and 0.740 for SHL-SRR, respectively).

Previous studies have shown that predictive accuracy increases with an increase in the RP size [11, 15, 16]. Our results are in line with previous findings with ∼10 to 35 fold increase in *R*^2^ using only markers when the RP size was increased from 75 to 8,000. Meuwissen [40], suggested the use of large RPs in estimation of marker effects, particularly for the GBLUP. However, in [30], the predictive accuracy of GLUP method was assessed using a small number of individuals (*n*_*R*_ of 5,300) relative to the 300,000 markers fitted in the model. Even though there are many other factors influencing predictive accuracy, a possible explanation of the low *R*^2^ given in [30] might be the small RP size. When *n*_*R*_ was 75 and the number of markers to be estimated was 4,200, predictive accuracies in scenarios SHL and SHR were low (0.017 in SHL, and 0.064 in SHR) as in the corresponding MAF scenarios in [30] for GENOVA data set. However, as mentioned above, predictive accuracy increased with the inclusion of more individuals in RP.

In derivation of the upper bound for *R*^2^ in [30], the conditional expectation of genetic values of VP individuals was written as

$$\begin{array}{c} E\left(u_{V_{i}}|{\text{y}}_{R}\right)={\text{g}}_{Q,i}{\left[{\text{G}}_{Q}\sigma_{u}^{2}+\text{I}\sigma_{e}^{2}\right]}^{-1}{\text{y}}_{R}, \end{array}$$ (7)

where **G**_*Q*_ is the relationship matrix of RP at the QTL level, and **y**_*R*_ is the vector of centered phenotypes of RP. The QTL genotypes of individuals are not known in reality, therefore genomic relationships are computed from marker genotypes instead of QTL. Thus, the conditional expectation is approximated as

$$\begin{array}{c} E\left(u_{V_{i}}|{\text{y}}_{R}\right)\approx {\text{g}}_{M,i}\left[{\text{G}}_{M}\sigma_{u}^{2}+\text{I}\sigma_{e}^{2}\right]{}^{-1}{\text{y}}_{R}, \end{array}$$ (8)

where **G**_*M*_ is the relationship matrix of RP at the marker level. In the derivation of an upper bound for *R*^2^ when this approximation is used, however, it was assumed that **G**_*Q*_ was known, and **g**_*M*,*i*_ was written as **g**_*M*,*i*_ = *b*_*i*_ **g**_*Q*,*i*_ + **ϵ**_*i*_ [30]. Then, as explained in [30], the approximation can be expressed as

$$\begin{array}{c} E\left(u_{V_{i}}|{\text{y}}_{R}\right)\approx b_{i}{\text{g}}_{Q,i}\left[{\text{G}}_{Q}\sigma_{u}^{2}+\text{I}\sigma_{e}^{2}\right]{}^{-1}{\text{y}}_{R}. \end{array}$$ (9)

In a population of unrelated individuals, the expected value of genomic relationships will be zero. When genomic relationships, **g**_*M*,*i*_ and **g**_*Q*,*i*_, are computed using 300,000 markers and 5,000 QTL as in [30], **g**_*Q*,*i*_ will have a much larger variance than **g**_*M*,*i*_. This results in the slope, *b*, of the regression of **g**_*M*,*i*_ on **g**_*Q*,*i*_ to be small. Thus, Eq (9) will have a much lower *R*^2^ than Eq (7). This would not be the case if Eq (8) was used, where both **g**_*Q*,*i*_ and **G**_*Q*_ are replaced with their marker based counterparts. This can be demonstrated by writing **g**_*M*,*i*_ = *b* **g**_*Q*,*i*_ and **G**_*M*_ = *b* **G**_*Q*_, where *b* is the average value of *b*_*i*_. Then the approximation Eq (8) becomes

$$\begin{array}{c} E\left(u_{V_{i}}|{\text{y}}_{R}\right)\approx {\text{g}}_{Q,i}\left[{\text{G}}_{Q}\sigma_{u}^{2}+\text{I}\frac{\sigma_{e}^{2}}{b}\right]{}^{-1}{\text{y}}_{R}. \end{array}$$ (10)

This approximation Eq (10) for the conditional expectation is almost identical to Eq (7), and therefore, the *R*^2^ from Eq (10) will be similar to the *R*^2^ from Eq (7).

### Comparison of Different Methods of Genomic Prediction

**Fig 2:**
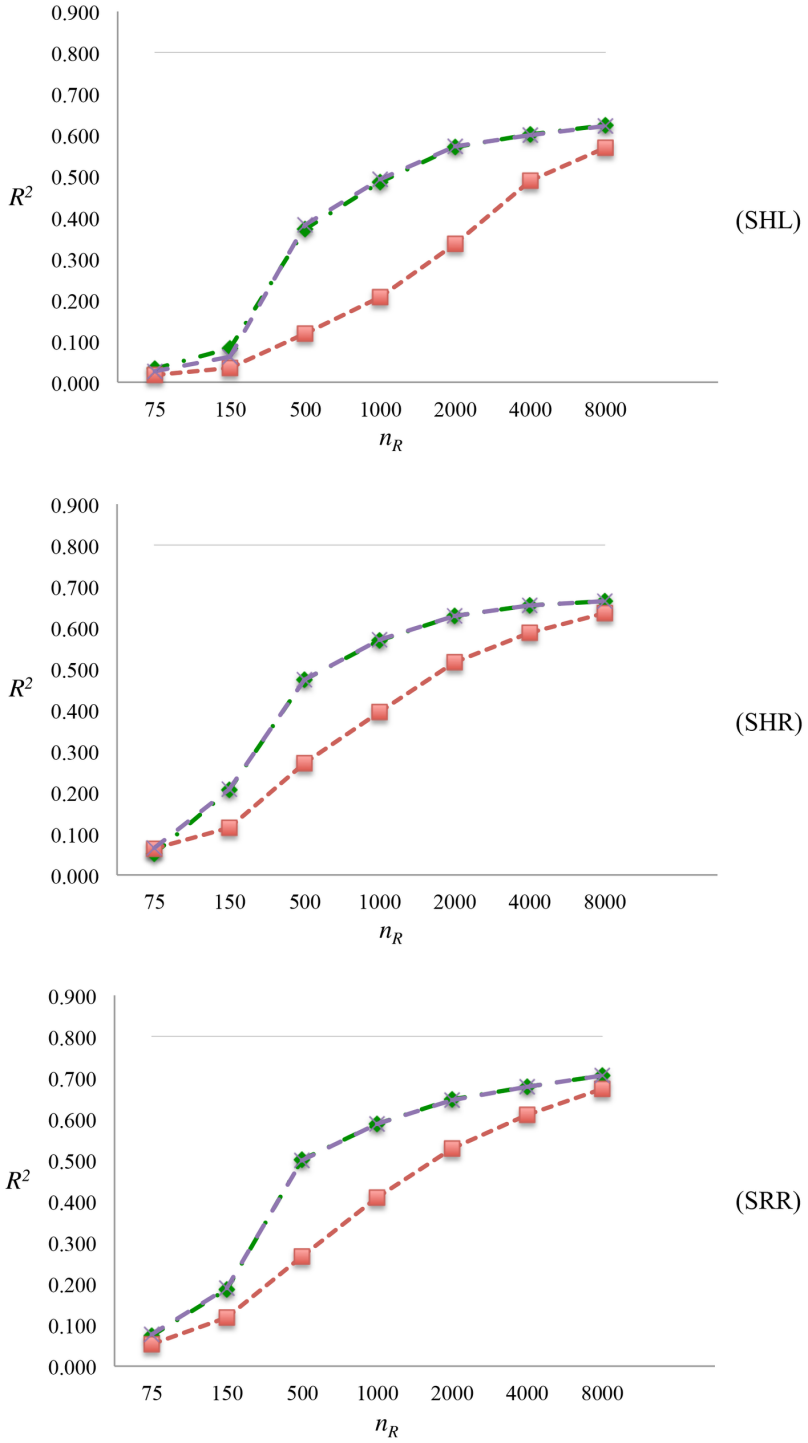
Comparison of different methods. SHL: Markers and QTL were selected for high and low MAFs, respectively; SHR: Markers were selected for high MAF, whereas QTL were selected at random; SRR: Markers and QTL were selected at random; *n*_*R*_: number of individuals in RP; *R*^2^: squared correlation between *y* and $\hat{u}$; simulated heritability, $h_{sim}^{2}$, (grey line); prediction *R*^2^s of GBLUP (red line), BayesB (green line) and BayesC (purple line).

Fig 2 shows *R*^2^s of GBLUP, BayesB and BayesC methods for varying values of *n*_*R*_ in all the scenarios when only the markers are in the panel. When *n*_*R*_ = 75, all methods had a similar *R*^2^, i.e., the variable selection methods, BayesB and BayesC, had no advantage over GBLUP. As *n*_*R*_ increased, initially, variable selection methods became superior to GBLUP, but eventually all methods yielded similar *R*^2^ values when *n*_*R*_ = 8,000.

**Fig 3:**
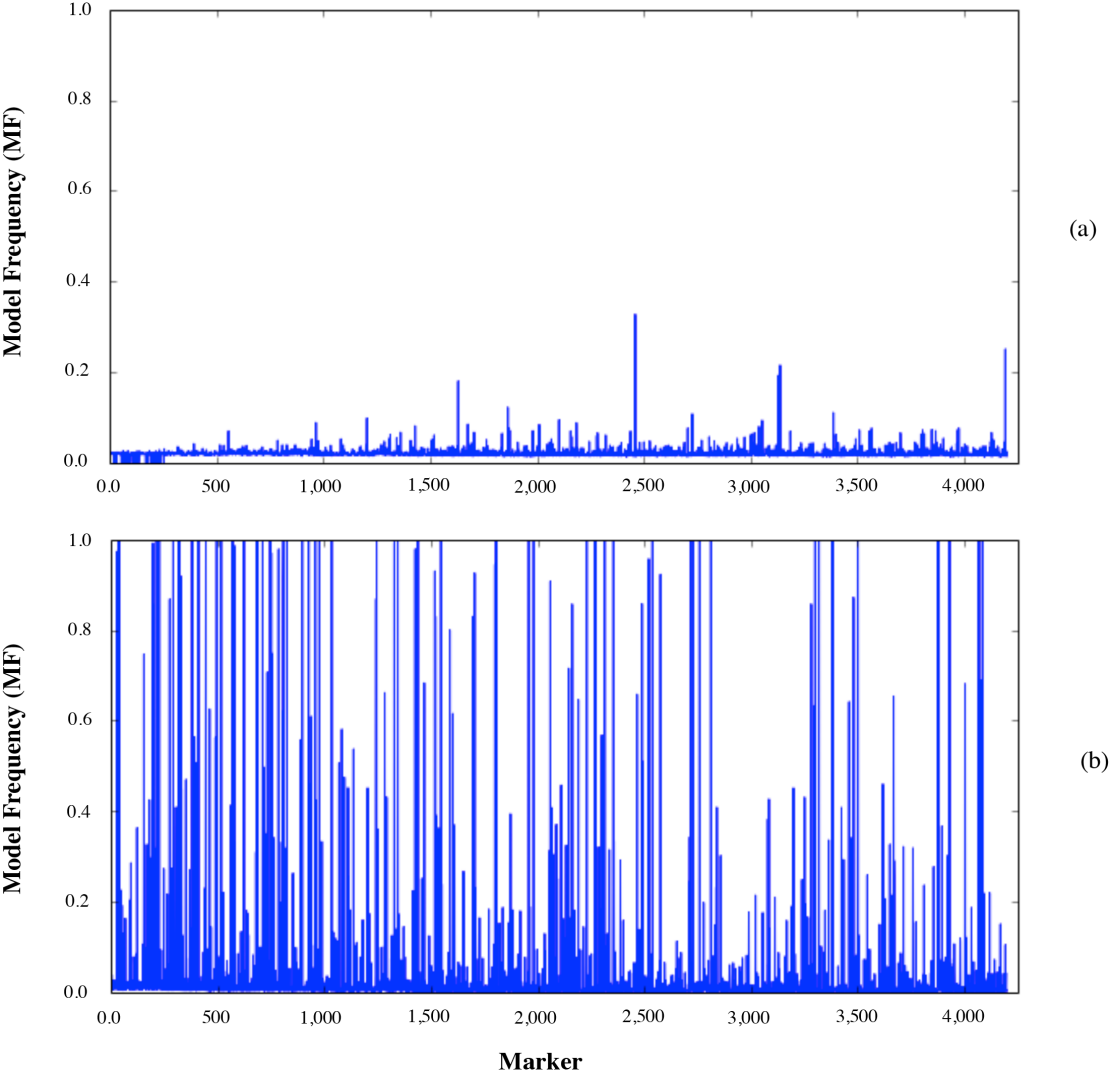
Model frequencies of markers in one replicate of SHL for BayesB method. SHL: Markers and QTL were selected for high and low MAFs, respectively; (a) *n*_*R*_ = 75; (b) *n*_*R*_ = 8,000.

For a given *n*_*R*_ and *h*^2^, predictive accuracy of GBLUP were shown to be highly dependent on *M*_*e*_, whereas predictive accuracy of BayesB is also dependent on *n*_*QTL*_ [11]. An approximation for the reliability of GP with BayesB was suggested with the modification of the equation in [28], which is given as *n*_*R*_ *h*^2^/[*n*_*R*_ *h*^2^ + *min*(*n*_*QTL*_, *M*_*e*_)] [11]. When *n*_*QTL*_ < *M*_*e*_, the advantage of variable selection methods, BayesB and BayesC, is expected to be more apparent since they select a subset of loci with an effect on the trait of interest instead of estimating the *M*_*e*_ parameters regardless of whether they have an effect [11]. Using the formula in [11], for *n*_*R*_ = 75, *h*^2^ = 0.8, *n*_*QTL*_ = 70 and *M*_*e*_ = 1,448, the predicted values of *R*^2^ were 0.370 and 0.032 for BayesB and GBLUP, respectively. However, the observed predictive accuracies for BayesB and BayesC in scenarios SHL to SRR were much lower (0.027-0.077) than these predicted values when *n*_*R*_ = 75. Fig 3 depicts the MFs of markers in one replicate of SHL for BayesB method when *n*_*R*_ = 75 and *n*_*R*_ = 8,000. It is clear that when *n*_*R*_ = 75 MFs followed a uniform distribution with none of the markers having MF higher than 0.4. On the other hand, there are many such markers 0.4 < MF when *n*_*R*_ = 8,000, and as *n*_*R*_ increased from 75 to 8,000, variance of the MF of markers increased more than 100-fold from 1.21 × 10^−4^ to 214 × 10^−4^ indicating that when *n*_*R*_ is small, variable selection does not effectively discriminate between markers that are in LD with QTL from those that are not. Therefore, one can not take full advantage of variable selection methods if *n*_*R*_ is not sufficiently large. An increase in *n*_*R*_ from 75 to 8,000 resulted in about a 10- to 20-fold increase in *R*^2^ for BayesB and BayesC (Tables 2–4). On the other hand, the advantage of variable selection methods over GBLUP diminished when *n*_*R*_ = 8,000, and all methods yielded similar predictive accuracies for this high heritability trait. Variable selection methods shrink marker effects that are very small towards zero, and therefore, these loci do not contribute to the estimation of *u*. However, when *π* of BayesC is set to zero, effects of all markers are estimated regardless of their size, which usually add only noise to the estimation of *u*. On the other hand, when sufficiently large RP is used, the effect of those markers that are very small can be estimated accurately, yielding a high predictive accuracy. This can explain why predictive accuracy from variable selection methods are higher than GBLUP when number of markers are much larger than *n*_*R*_, and why eventually all methods yielded same predictive accuracies.

When the QTL were included in the panel, the gap between predictive accuracies of GBLUP and BayesB or BayesC was higher than when only markers were in the panel (Tables 2–4). Moreover, in this case, BayesB and BayesC with relatively small values of *n*_*R*_ (500-1,000) could achieve the predictive accuracy of GBLUP with *n*_*R*_ = 8,000.

It was reported [41] that the predictive accuracy of BayesB, was not greater than that of GBLUP, which we believe is due to the circumstance that the number of markers fitted (∼2.5 million) greatly exceeded the small number of RP, *n*_*R*_ = 155. Several studies have investigated the effect of different methods on predictive accuracy [10–12, 24, 40, 42], and it can be concluded that none of these methods is universally best, and the performance of the method depends on the genetic architecture and the heritability of the trait as well as the RP size.

### Realized Relationships and Estimability

We examined the effect of relationship on predictive ability using the maximum realized relationship of individuals in VP, *max*(**g**_*V*_*i*__), to the individuals in RP for two extreme groups of individuals, those with low relationship (L) or high relationship (H). Table 5 summarizes the predictive abilities obtained by GBLUP for L and H groups at varying RP sizes and for two quantitative traits with *h*^2^ = 0.8 and 0.999. An important aspect of Table 5 is that, when RP size, *n*_*R*_, increased, the number of individuals (*n*_L_) with *max*(**g**_*V*_*i*__)<0.15 decreased, while the number of individuals *n*_H_ with *max*(**g**_*V*_*i*__)≥0.25 increased. In the scenario where *h*^2^ = 0.8, when *n*_*R*_ = 500 and *n*_*R*_ = 5,000 there were 1,347 and 163 individuals in L group, while there were 8 and 69 individuals in H group. The trends in the number of individuals in both L and H groups indicates that when more individuals are included in RP, the probability of having at least one individual in the RP with a high relationship for a VP individual increases. However, even when *n*_*R*_ was 5,000, among the 2,000 VP individuals there were still only 69 and 64 of 2,000 individuals in H group for *h*^2^ = 0.8 and *h*^2^ = 0.999, respectively.

**Table 5:** Predictive accuracies and estimabilities for relationship groups.

*h*^2^	*n*_*R*_	*n*_*V*_	*n*_*L*_	*n*_*H*_	$R_{L}^{2}$	$R_{H}^{2}$	${\bar{\mathrm{UP}}}_{L}$	${\bar{\mathrm{UP}}}_{H}$
0.8	500	2,000	1347	8	0.352(0.012)	0.439(0.083)	0.549(0.000)	0.569(0.003)
	1,000	2,000	972	17	0.477(0.006)	0.469(0.050)	0.777(0.000)	0.791(0.001)
	2,000	2,000	546	31	0.614(0.009)	0.614(0.043)	0.942(0.000)	0.947(0.000)
	5,000	2,000	163	69	0.699(0.011)	0.733(0.017)	1.000(0.000)	1.000(0.000)
0.999	500	2,000	1342	8	0.542(0.011)	0.533(0.080)	0.550(0.000)	0.575(0.002)
	1,000	2,000	963	15	0.756(0.005)	0.704(0.047)	0.777(0.000)	0.792(0.001)
	2,000	2,000	558	29	0.932(0.002)	0.939(0.008)	0.942(0.000)	0.948(0.000)
	5,000	2,000	166	64	0.995(0.000)	0.996(0.000)	1.000(0.000)	1.000(0.000)

*h*^2^: heritability of the trait; *n*_*R*_: number of individuals in RP; *n*_*V*_: number of individuals in VP; *n*_*L*_ and *n*_*H*_ are the average number individuals in L and H groups over replicates, respectively; $R_{L}^{2}$ and $R_{H}^{2}$ are the predictive accuracies in L and H groups, respectively; ${\bar{UP}}_{L}$ and ${\bar{UP}}_{H}$ are the mean of the average *UP* at each replicate for L and H, respectively.

Predictive ability tended to increase not only for H group but also for L group individuals as the *n*_*R*_ increased (Table 5). For *h*^2^ = 0.8, predictive abilities in L group, $R_{L}^{2}$, were 0.352, 0.477, 0.614 and 0.699, while predictive abilities in H group, $R_{H}^{2}$, were 0.439, 0.469, 0.614 and 0.733 when *n*_*R*_ was 500, 1,000, 2,000 and 5,000, respectively. For *h*^2^ = 0.999, Predictive abilities in L group, $R_{L}^{2}$, were 0.542, 0.756, 0.932 and 0.995, while in H group $R_{H}^{2}$ were 0.533, 0.704, 0.939 and 0.996 when *n*_*R*_ was 500, 1,000, 2,000 and 5,000, respectively. This implies that even when the pairwise relationships between VP and RP individuals are low, one can obtain high predictive ability.

We approached GP as an estimability problem, and derived an upper bound for reliability, and thus the upper bound for *R*^2^. Mean values of upper bound for reliability, $\bar{UP}$, are also given in Table 5 for L and H groups. When multiplied by the trait heritability, the upper bound for *R*^2^ is obtained, $h^{2}\bar{UP}$. For *h*^2^ = 0.8, the upper bounds of $R_{L}^{2}$ were 0.439, 0.622, 0.754 and 0.800, whereas the upper bounds of $R_{H}^{2}$ were 0.455, 0.633, 0.758 and 0.800. When *h*^2^ = 0.999, the upper bounds for $R_{L}^{2}$ were 0.549, 0.776, 0.941 and 0.999, whereas the upper bounds for $R_{H}^{2}$ were 0.574, 0.791, 0.947 and 0.999.

Our results indicate that predictive ability depends on how well an individual’s genotype vector in VP can be written as a linear combination of the rows of the genotype matrix of RP individuals. As *n*_*R*_ increases, the row space of **X**_*R*_ will tend to increase and the possibility that **x**_*V*_ is in the row space of **X**_*R*_ will also increase. Based on these results, it can be concluded that prediction *R*^2^ is limited by $h^{2}\bar{UP}$.

Habier et al. [9, 12], showed that a high relationship between the individuals in VP and RP resulted in a high predictive ability using simulated and real data. Legarra et al. [34], reported a higher predictive ability within-family than across-family in mice. Clark et al. [33], concluded that the overall prediction of breeding values relied on the degree of relationship between the VP and RPs. Pszczola et al. [13], examined the predictive ability for varying levels of relationships within RP, and between VP and RP. Their results also showed that to achieve a high predictive ability, a high relationship is required between VP and RP. Makowsky et al. [15], showed that predictive ability increases with an increase in the number of close relatives of VP individuals in the RP. On the other hand, Luan et al. [10] investigated predictive ability of GP for a dairy cattle breed, and their findings indicated an important aspect of the relationship between RP and VPs. Contrary to the above-mentioned studies, their results did not provide any strong evidence for the effect of relationship between RP and VP. In this study, we have shown that provided that the genotypes of VP individuals are in the row space of **X**_*R*_, high predictive ability can be obtained depending on the heritability of the trait and the RP size even when the pairwise relationships between VP and RP are low. This is consistent with the suggestion by Calus [43] that use of a RP comprising the whole range of phenotypes and genotypes is the requirement to obtain reliable predictions.

## Supporting Information

S1 Text Includes the derivations to reach the approximation for *R*^2^ given as Eq (1), and derivations leading to the upper bound of *R*^2^ presented in the manuscript. (PDF)
