# Supplementary material for: An Upper Bound for Accuracy of Prediction Using GBLUP
Source: PLoS One. 2016 Aug 16;11(8):e0161054. doi: 10.1371/journal.pone.0161054 (PMC4986954; doi:10.1371/journal.pone.0161054)
Supplement: S1 Text — (PDF) [file pone.0161054.s001.pdf]

Appendix 1

Following Goddard et al. [1], reliability of GP can be approximated with

$$r^2 \approx q^2 \frac{\theta}{\theta + 1} \tag{1}$$

where $\theta = n_R q^2 h^2 / M_e$ and q^2 is the proportion of genetic variance explained by markers, $q^2 = \sigma_q^2/\sigma_u^2$. Substitution of θ in (1) yields:

$$r^2 \approx q^2 \frac{n_R q^2 h^2}{n_R q^2 h^2 + M_e}, \tag{2}$$

or

$$r^2 \approx q^2 \frac{n_R h^2}{n_R h^2 + \frac{M_e}{q^2}}.$$

as given in the text. Since $h^2 = \sigma_u^2/\sigma_y^2$ and $q^2 = \sigma_q^2/\sigma_u^2$, we can re-write equation (2) as:

$$r^2 \approx q^2 \frac{n_R h_M^2}{n_R h_M^2 + M_e}, \tag{3}$$

where h_M^2 is the genomic heritability, $h_M^2 = \sigma_q^2/\sigma_y^2$ [2].
Because $R^2_{\hat{u},y} = \frac{Cov(\hat{u},y)^2}{Var(\hat{u})Var(y)}$ and $r^2_{\hat{u},u} = \frac{Cov(\hat{u},u)^2}{Var(\hat{u})Var(y)h^2}$, then $R^2_{\hat{u},y} = h^2 r^2_{\hat{u},u}$. Therefore, when the reliabilities are computed as the squared correlation between $\hat{u}$ and y, (3) has to be multiplied by h^2 to be comparable. Doing so yields equation (1) in the main text:

$$R^2 \approx h_M^2 \frac{n_R h_M^2}{n_R h_M^2 + M_e}$$

and

$$lim_{n_R \to \infty} h_M^2 \left(\frac{n_R h_M^2}{n_R h_M^2 + M_e} \right) = h_M^2$$

Appendix 2

In the following section, we will use the same notation as in the main text to denote the genotype matrix of RP individuals and vector of genotypes of VP individuals, and show that $\tilde{\boldsymbol{\beta}} \in \Re(\mathbf{X}_R)$.
Consider the fixed linear model:

$$\mathbf{y} = \mathbf{X}\boldsymbol{\beta} + \mathbf{e},$$

where $\mathbf{y}$ is the $n \times 1$ vector of observations corrected for the mean, $\mathbf{X}$ is the $n \times p$ matrix of centered genotypes, $\boldsymbol{\beta}$ is the $p \times 1$ vector of random marker effects with null mean and covariance matrix $\mathbf{I}\sigma_\beta^2$, and $\mathbf{e}$ is the $n \times 1$ vector of random

environmental effects with null mean and covariance matrix $\mathbf{I}\sigma_e^2$. The BLUP of $\boldsymbol{\beta}$ is the solution to:

$$(\mathbf{X}_R'\mathbf{X}_R + \mathbf{I}\lambda)\tilde{\boldsymbol{\beta}} = \mathbf{X}_R'\mathbf{y}_R, \tag{4}$$

where $\lambda = \frac{\sigma_e^2}{\sigma_\beta^2}$. In general, it is possible to write $\tilde{\boldsymbol{\beta}}$ as

$$\tilde{\boldsymbol{\beta}} = \mathbf{b}_1 + \mathbf{b}_2, \tag{5}$$

where $\mathbf{b}_1 \in \Re(\mathbf{X}_R)$ and $\mathbf{b}_2$ is orthogonal to $\Re(\mathbf{X}_R)$ [3]. Then using (5), (4) can be written as

$$(\mathbf{X}_R'\mathbf{X}_R\mathbf{b}_1 + \mathbf{I}\lambda\mathbf{b}_1) + (\mathbf{X}_R'\mathbf{X}_R\mathbf{b}_2 + \mathbf{I}\lambda\mathbf{b}_2) = \mathbf{X}_R'\mathbf{y}_R \tag{6}$$

Because $\mathbf{b}_1 \in \Re(\mathbf{X}_R)$, $(\mathbf{X}_R'\mathbf{X}_R\mathbf{b}_1 + \mathbf{I}\lambda\mathbf{b}_1) \in \Re(\mathbf{X}_R)$, and because $\mathbf{b}_2$ is orthogonal to $\Re(\mathbf{X}_R)$ and $\mathbf{X}_R'\mathbf{X}_R \in \Re(\mathbf{X}_R)$, $\mathbf{X}_R'\mathbf{X}_R\mathbf{b}_2 = 0$. So, (6) can be rearranged as:

$$\mathbf{I}\lambda\mathbf{b}_2 = \mathbf{X}_R'\mathbf{y}_R - \mathbf{X}_R'\mathbf{X}_R\mathbf{b}_1 - \mathbf{I}\lambda\mathbf{b}_1 \tag{7}$$

Since $\mathbf{X}_R'\mathbf{y}_R \in \Re(\mathbf{X}_R)$, $\mathbf{X}_R'\mathbf{X}_R \in \Re(\mathbf{X}_R)$ and $\mathbf{b}_1 \in \Re(\mathbf{X}_R)$, the right hand side of (7) is in $\Re(\mathbf{X}_R)$. Further, the left hand side of (7) is orthogonal to $\Re(\mathbf{X}_R)$ by construction. Thus $\mathbf{b}_2$ is in $\Re(\mathbf{X}_R)$ but is also orthogonal to $\Re(\mathbf{X}_R)$. This means $\mathbf{b}_2$ is orthogonal to itself, which can only be true if $\mathbf{b}_2 = \mathbf{0}$. It follows that $\tilde{\boldsymbol{\beta}} = \mathbf{b}_1$ which is in $\Re(\mathbf{X}_R)$.

Appendix 3

In this section we are going to derive an upper bound (UP_i) for the reliability of $\hat{u}_i$:

$$Cor^2(u_i, \hat{u}_i) = \frac{Var(\hat{u}_i)}{Var(u_i)},$$

from which an upper bound of R^2 can also be obtained. Recall that $\hat{u}_i = \mathbf{x}_{V_1}'\tilde{\boldsymbol{\beta}}$. So, the prediction error variance for $\hat{u}$ can be written as [4]

$$\begin{aligned} Var(u_i - \hat{u}_i) &= Var(u_i) - Var(\hat{u}_i) \\ &= \mathbf{x}_{V_1}'(\mathbf{X}_R'\mathbf{X}_R + \mathbf{I}\lambda)^{-1}\mathbf{x}_{V_1}\sigma_e^2. \end{aligned}$$

Then, it follows that,

$$\begin{aligned} Var(\hat{u}_i) &= Var(\mathbf{x}_{V_1}'\tilde{\boldsymbol{\beta}}) \\ &= Var(\mathbf{x}_{V_1}'\boldsymbol{\beta}) - \mathbf{x}_{V_1}'(\mathbf{X}_R'\mathbf{X}_R + \mathbf{I}\lambda)^{-1}\mathbf{x}_{V_1}\sigma_e^2. \end{aligned}$$

From the above equation, it can be seen that when $h^2 \to 1$, $\sigma_e^2 \to 0$ and $Var(\mathbf{x}_{V_1}'\tilde{\boldsymbol{\beta}}) \to Var(\mathbf{x}_{V_1}'\boldsymbol{\beta})$. Assuming $Var(\boldsymbol{\beta}) = \mathbf{I}\sigma_\beta^2$, $Var(\mathbf{x}_{V_1}'\boldsymbol{\beta}) = \mathbf{x}_{V_1}'\mathbf{x}_{V_1}\sigma_\beta^2$, and thus

$$UP_i = \frac{\mathbf{x}'_{V_1}\mathbf{x}_{V_1}}{\mathbf{x}'_V\mathbf{x}_V}$$

is an upper bound for reliability, and therefore $h^2 UP_i$ is an upper bound for R^2. Note that when $h^2 = 1$, then $\lambda = 0$, and therefore, $\tilde{\boldsymbol{\beta}}$ is identical to the least square estimator of $\boldsymbol{\beta}$, and will no longer be in $\Re(\mathbf{X}_R)$.

References

[1] ME Goddard, BJ Hayes, and THE Meuwissen. Using the genomic relationship matrix to predict the accuracy of genomic selection. *J Anim Breed Genet*, 128:409–421, 2011.

[2] G de los Campos, AI Vazquez, RL Fernando, YC Klimentidis, and D Sorensen. Prediction of complex human traits using the genomic best linear unbiased predictor. *PLoS Genet*, 9(7):e1003608, 2013.

[3] Henry Scheffe. *The analysis of variance*, volume 72. John Wiley & Sons, 1999.

[4] CR Henderson. Applications of linear models in animal breeding. *Guelph, Ontario, Canada: Univ. Guelph.*, 1984.
